# Phytochemical Investigation of *Cordia africana* Lam. Stem Bark: Molecular Simulation Approach

**DOI:** 10.3390/molecules27134039

**Published:** 2022-06-23

**Authors:** Manal M. Sabry, Ahlam M. El-Fishawy, Ahmed A. El-Rashedy, Rania A. El Gedaily

**Affiliations:** 1 Department of Pharmacognosy, Faculty of Pharmacy Cairo University, Cairo 11562, Egypt; ahlam.elfishawy@pharma.cu.edu.eg (A.M.E.-F.); rania.elgedaily@pharma.cu.edu.eg (R.A.E.G.); 2 Natural and Microbial Products Department, National Research Center (NRC), Giza 12622, Egypt; ahmedelrashedy45@gmail.com

**Keywords:** *Cordia africana* stem bark, HPLC, rosmarinic acid, methyl rosmarinate, antibacterial activity, molecular dynamic

## Abstract

Background: The current work planned to evaluate *Cordia africana* Lam. stem bark, a traditionally used herb in curing of different ailments in Africa such as gastritis and wound infections, based on phytochemical and antibacterial studies of two pathogenic microorganisms: methicillin-resistant *Staphylococcus aureus* (MRSA) and *Helicobacter pylori*. Methods: High performance liquid chromatography (HPLC) profiling was used for qualitative and quantitative investigation of the ethanol extract. The minimum inhibitory concentration (MIC) of the ethanolic extract and isolated compounds was estimated using the broth microdilution method and evidenced by molecular dynamics simulations. Results: Four compounds were isolated and identified for the first time: α-amyrin, *β*-sitosterol, rosmarinic acid (RA) and methyl rosmarinate (MR). HPLC analysis illustrated that MR was the dominant phenolic acid. MR showed the best bacterial inhibitory activity against MRSA and *H. pylori* with MIC 7.81 ± 1.7 μg/mL and 31.25 ± 0.6, respectively, when compared to clarithromycin and vancomycin, respectively. Conclusion: The antibacterial activity of the stem bark of *Cordia africana* Lam. was evidenced against MRSA and *H. pylori*. Computational modeling of the studied enzyme-ligands systems reveals that RA and MR can potentially inhibit both MRSA peptidoglycan transpeptidases and *H. pylori* urease, thereby creating a pathway via the use of a double target approach in antibacterial treatment.

## 1. Introduction

From an ethnobotanical point of view, *Cordia africana* Lam. is one of the most widely recognized indigenous plants in Ethiopia and Sudan [1]. It is used for traditional treatment of different ailments such as liver disease, amoebiasis, stomachache, gastritis, diarrhea, cough and wounds [2,3]. The leaf and stem bark are the most frequently used parts in treatment [1]. In Nigeria, the stem bark is utilized as an antispasmodic and anti-inflammatory, while in East Africa, it is employed to treat wounds, skin infections and gastritis [4]. MRSA plays a chief role in the epidemiology and pathogenesis of infections in humans, especially in the skin and soft tissues [5,6]. Gastritis is always expected in cases of *H. pylori* infections, which affect 70.1% of the populations in Africa [7].

Few phytochemical investigations have been directed predominantly to the seed oil characterization [8] and determination of the total phenolics content of the fruit [9], as well as the isolation of quercetin, *p*-hydroxy phenyl lactic acid, isorhamnetin-3-*O*-rutinoside, quercetin-3-*O*-β-D-glucopyranoside, gallic acid and rutin from the ethanolic leaf extract [10].

Concerning the pharmacological effects of *Cordia africana* Lam, the leaf and the stem bark showed antioxidant, anti-inflammatory, cytotoxic and antibacterial activities against *Mycobacterium smegmatis*, *M. aurum*, *M. fortuitum*, *Bacillius cereus*, *Staphylococcus aureus*, *Enterococcus faecalis*, *Pseudomonas aeruginosa*, and *Salmonella typhimurium* [11,12], in addition to *Streptococcus pyogenes*, *Escherichia coli* and *Klebsiella pneumonia* [13]. The stem bark ethanolic extract exhibited anti-nociceptive [4] and anti-diabetic effects [10].

*Staphylococcus aureus* is a pathogenic *Gram*-positive bacterium that plays a chief role in the epidemiology and pathogenesis of infections in humans, especially in the skin and soft tissues [5,6]. Methicillin-resistant *S. aureus* (MRSA) strains were genetically modified to be resistant to all *β*-lactam antibiotics [5]. This resistance is mediated through the formation of penicillin-binding proteins. It is estimated that the number of people who die from MRSA infections exceeds that of other life-threating diseases such as the acquired immunodeficiency syndrome (AIDS) and tuberculosis [14]. *β*-lactam antibiotics act through irreversible inhibition of transpeptidases, known as penicillin-binding proteins (PBPs) that catalyze the formation of peptidoglycan in the bacterial cell wall [15]. A broad resistance is created by MRSA to β-lactams through the manufacture of penicillin-binding protein 2a and the possession of a gene cassette with mecA, encoding the modified, low affinity transpeptidase, PBP2a [16]. Vancomycin is the most frequent antibiotic used in the treatment of MRSA infections [17].

*Helicobacter pylori* is a pathogenic *Gram*-negative bacilliform bacterium colonizing the stomach of more than 50% of human beings on Earth [18]. The prevalence of the infection in Africa is 70.1% of the population [7]. It is considered one of the causes of peptic ulcers related to a high risk of gastric adenocarcinoma [18,19]. Despite the lack of symptoms in most cases, chronic inflammation and gastritis should always be expected [20,21]. *H. pylori* is life-threatening only in a minority of patients who develop gastric ulcers, carcinoma and autoimmune inflammation, or *β*-cell lymphoma [22]. To be able to persist to the acidic pH condition of the gastric juice, *H. pylori* produces high concentrations of urease enzymes that hydrolyze the urea to ammonia, which buffers the gastric environment [23]. The urease acts as a ligand to induce hypoxia-induced factor-1α, which is commonly related to the growth of various types of cancer [23]. Clarithromycin is the antibiotic of choice for this bacterium as it blocks the transpeptidation and the translocation reactions of *H. pylori* and hence inhibits its protein synthesis [24].

The need for an effective therapy prompted the advancement of many drug combination schemes involving amoxycillin, clarithromycin and/or metronidazole with any proton pump inhibitor [25], although repeated use of these drugs was associated with resistance and undesirable effects on intestinal flora [26]. Many plant-derived extracts and their isolated secondary metabolites have been proven to possess anti-*H. pylori* activity [27,28,29]. This emphasizes the urgent need to search for novel active antibacterial agents against these two pathogenic bacteria.

The current investigation aimed to estimate the phytochemistry and the antibacterial activity of *Cordia africana* Lam. stem bark and its main isolated compounds against two pathogenic microorganisms, methicillin-resistant *S. aureus* (MRSA) and *H. pylori,* as possible causative agents for wound inflammation and gastritis, respectively. This study was performed for the first time for this plant to help the rationalization of its traditional use using the molecular docking technique.

## 2. Results

### 2.1. Phytochemical Analysis

#### 2.1.1. Total Phenolic Acid and Flavonoid Content

Total phenolic acid content was 95.17 ± 0.1 µg (GAE)/mg dried ethanolic extract, while the total flavonoid content was 16.11 ± 0.05 µg (QE)/mg dried ethanolic extract.

#### 2.1.2. Characterization of Isolated Compounds

Data of compound 1: ^1^H-NMR (CDCl_3_, 400 MHz): 4.43 (1H, *t*, H-3), 5.14 (1H, *d*, H-12), 0.93 (3H, *s*, H-23), 0.73 (3H, *s*, H-24), 0.80 (3H, *s*, H-25), 0.77 (3H, *s*, H-26), 1.1 (3H, *s*, H-27), 1.05 (3H, *s*, H-28), 0.87 (3H, *d*, H-29), 0.84 (3H, *d*, H-30), and 1.28–2.15 ppm (remaining 23 protons). ^13^C-NMR (CDCl_3_, 100 MHz): 140.1 (C-13), 125.0 (C-12), 79.4 (C-3), 59.5 (C-18), 55.7 (C-5), 48.2 (C-9), 43.4 (C-14), 41.1 (C-8), 41.0 (C-22), 40.3 (C-19), 39.0 (C-20), 38.6 (C-1), 38.0 (C-4), 37.4 (C-10), 37.4 (C-16), 33.2 (C-17), 31.7 (C-21), 29.1 (C-15), 28.5 (C-28), 28.0 (C-23), 24.0 (C-2), 23.8 (C-7), 23.8 (C-27), 23.5 (C-29), 21.5 (C-30), 19.0 (C-6), 18.2 (C-11), 16.9 (C-26), 16.8 (C-24) and 14.9 (C-25).

Data of compound 2: ^1^H-NMR (CDCl_3_, 400 MHz) δ: 3.56 (1H, *m*, H-3), 5.36 (1H, *m*, H-5), 0.66 (3H, *s*, H-28), 0.69 (3H, *d*, *J* = 7.12, H-26), 0.81 (3H, *d*, *J* = 8, H-24), 0.85 (3H, *d*, *J* = 7.68, H-26), 0.92 (3H, *d*, *J* = 6.4, H-19), 1.01 (3H, *s*, H-29). ^13^C-NMR (CDCl_3_, 100 MHz) 37.3 (C-1), 40.5 (C-2), 71.8 (C-3), 42.3 (C-4), 140.8 (C-5), 121.7 (C-6), 31.6 (C-7), 31.9 (C-8), 50.2(C-9), 36.5 (C-10), 21.2 (C-11), 39.8 (C-12), 42.3 (C-13), 56.9 (C-14), 26.1 (C-15), 28.3 (C-16), 56.1 (C-17), 36.2 (C-18), 19.1 (C-19), 33.9 (C-20), 25.4 (C-21), 45.8 (C-22), 23.1 (C-23), 12.3 (C-24), 29.2 (C-25), 19.8 (C-26), 19.4 (C-27), 19 (C-28), 12.0 (C-29).

Data of compound 3: ^1^H-NMR (CD_3_OD, 400 MHz) δ: 7.08 (1H, *d*, *J* = 2.0 Hz, H-2′), 6.81 (1H, *d*, *J* = 8.0 Hz, H-5), 6.98 (1H, *d*, *J* = 8.5 Hz, H-2), 7.57 (1H, *d*, *J* = 16.0 Hz, H-7), 6.27 (1H, *d*, *J* = 16.2 Hz H-8′), 6.73 (1H, *d*, *J* = 2.0 Hz, H-2), 6.74 (1H, *d*, *J* = 8.0 Hz, H-5′), 6.60 (1H, *dd*, *J* = 8.1, 2.1 Hz, H-6), 5.20 (1H, *dd*, *J* = 10.0, 3.5 Hz, H-8), 3.32 (1H, *dd*, *J* = 14.5, 5.5 Hz, H-7a), 3.07 (1H, *dd*, *J* = 14.5, 5.5 Hz, H-7b). ^13^C-NMR (CD_3_OD, 100 MHz): δ; 170.9 (C9), 167.1 (C-9′ ), 149.4 (C-4′), 145.7 (C-7′), 145.3 (C-3), 144.7 (C-4), 143.9 (C- 3′), 127.4 (C-1), 126.2 (C-1′), 121.9 (C-6), 120.5 (C-6′), 116.2 (C-2), 115.2 (C-5), 115.1 (C-5′), 114.0 (C-8′), 113.3 (C-2′), 73.3 (C-8), 36.5 (C-7).

Data of compound 4: ^1^H-NMR (CD_3_OD, 400 MHz): δ; 7.52 (1H, *d*, *J* = 15.5 Hz, H-7′), 7.04 (1H, *d*, *J* = 2.0 Hz, H-2′), 6.91 (H, *dd*, *J* = 8.5, 2.0 Hz, H-6′), 6.75 (1H, *d*, *J* = 8.5 Hz, H-5′), 6.71 (1H, *d*, *J* = 2.0 Hz, H-2), 6.64 (1H, *d*, *J* = 8.0 Hz, H-5), 6.61 (1H, *dd*, *J* = 8.0, 2.0 Hz, H-6), 6.27 (1H, *d*, *J* = 15.5 Hz, H8′), 5.11 (1H, *dd*, *J* = 7.5, 5.0 Hz, H-8), 3.72 (3H, *s*, OCH_3_), 3.06 (1H, *dd*, *J* = 14.5, 5.5 Hz, H-7a), 3.00 (1H, *dd*, *J* = 14.5, 5.5 Hz, H-7b); ^13^C-NMR (CD_3_OD, 100MHz): δ; 172.1 (C-9), 168.2 (C9′), 150.1 (C-4′), 148.6 (C-7′), 147.3 (C-3′), 146.1 (C-3), 145.7 (C-4), 128.4 (C-1), 127.5 (C-1′), 123.3 (C-6′), 122.8 (C-6), 117.9 (C-2), 116.9 (C-5), 116.2 (C-5′), 115.3 (C-2′), 114.2 (C-8′), 74.9 (C-8), 52.8 (OCH_3_), 38.0 (C-7).

#### 2.1.3. Identification of Compounds

**Figure 1:**
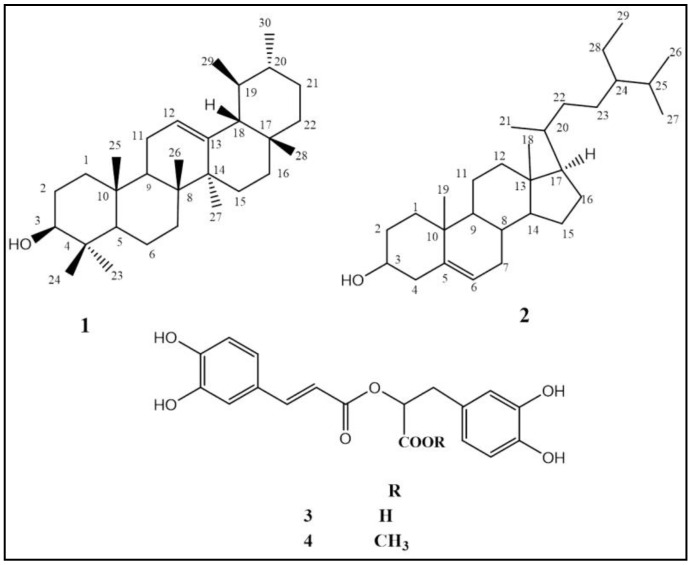
Chemical structures of isolated compounds from *Cordia africana* Lam.

Four compounds were isolated from *Cordia africana* Lam. stem bark for the first time and were identified as *α*-amyrin (1) and *β*-sitosterol (2) from the methylene chloride extract, and rosmarinic acid (RA) (3) and methyl-rosmarinate (MR) (4) from the *n*-butanol fraction. However, MR, RA [30,31], *α*-amyrin and *β*-sitosterol [32,33] were previously isolated from the genus. Identification of compounds was performed based on ^1^H-NMR and ^13^C-NMR in accordance with the literature [33,34]. The chemical structures of the isolated compounds from *Cordia africana* Lam. are illustrated in Figure 1.

#### 2.1.4. HPLC Analysis

**Figure 2:**
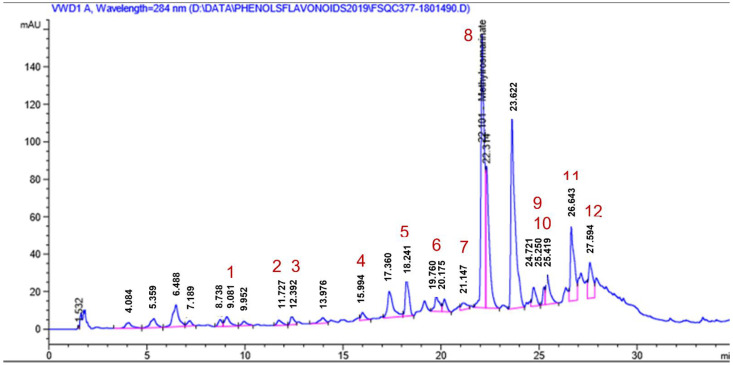
HPLC chromatogram at λ284 nm of the ethanolic extract of *Cordia africana* Lam. stem bark (2 mg/mL).

The occurrence of RA and MR was confirmed by HPLC analysis (Table 1) in which MR was the major identified phenolic acid (727.66 ppm) in addition to ten other phenolic compounds (Table 1) where *p*-hydroxy benzoic (124.68 ppm), caffeic (8.95 ppm), rosmarinic (138.18 ppm), *o*-coumaric (23.07 ppm), syringic (22.44 ppm) and ferulic (14.47 ppm) acids were present in appreciable amounts together with five flavonoids: naringenin, kaempferol, rutin and myricetin, which amounted to 1971.68, 943.38, 499.63 and 452.86 ppm, respectively. Quercetin was also present in an appreciable amount (239.75 ppm). The HPLC chromatogram is shown in Figure 2.

### 2.2. Antibacterial Activity

**Figure 3:**
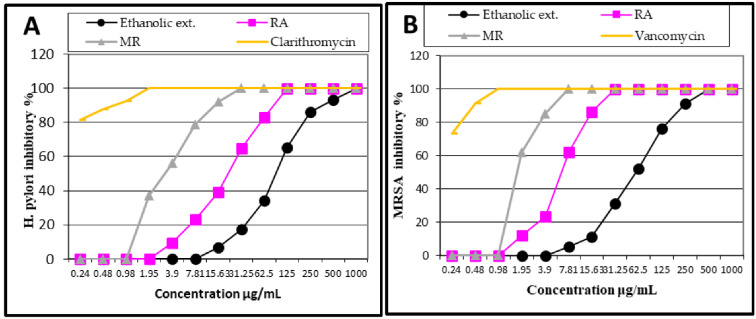
Dose response curves of stem bark ethanolic extract of *Cordia africana*, rosmarinic acid (RA), and methyl rosmarinate (MR) versus clarithromycin against (**A**): *H. pylori* and vancomycin against (**B**): methicillin-resistant *Staphylococcus aureus* (MRSA).

The obtained antibacterial results of the ethanolic extract and the two isolated compounds, RA and MR, against *Helicobacter pylori* and MRSA using the broth microdilution method are shown in Table 2; and the dose-response curves against the standards are illustrated in Figure 3. The results demonstrated that MR showed the best antibacterial inhibitory activity against MRSA and *H. pylori* with MIC 7.81 ± 1.7 and 31.25 ± 0.9 μg/mL, respectively, followed by RA compared to vancomycin (MIC 0.98 ± 1.6 μg/mL) and clarithromycin (MIC 1.95 ± 0.8 μg/mL).

### 2.3. Molecular Docking Analysis

#### 2.3.1. Molecular Dynamic and System Stability

**Figure 4:**
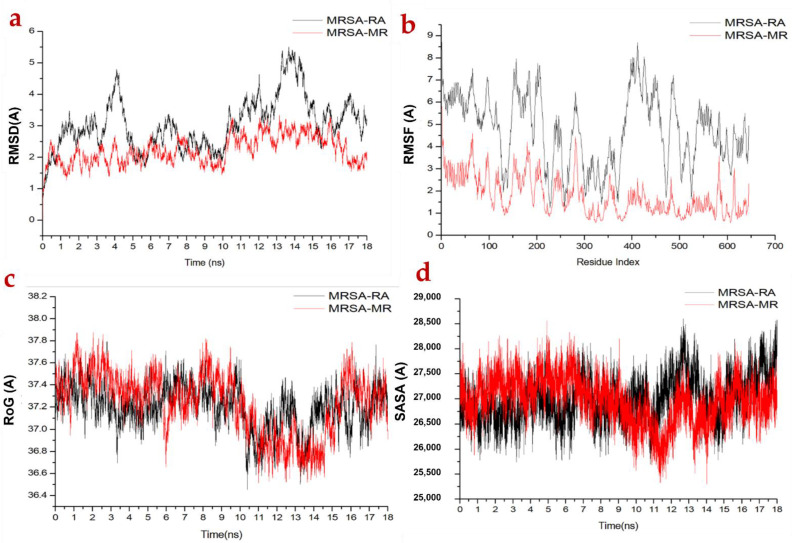
(**a**) RMSD of Cα atoms of the protein backbone atoms; (**b**) RMSF of each residue of the protein backbone Cα atoms; (**c**) ROG of Cα atoms of protein residues; (**d**) solvent accessible surface area (SASA) of the backbone atoms relative to the starting.

**Figure 5:**
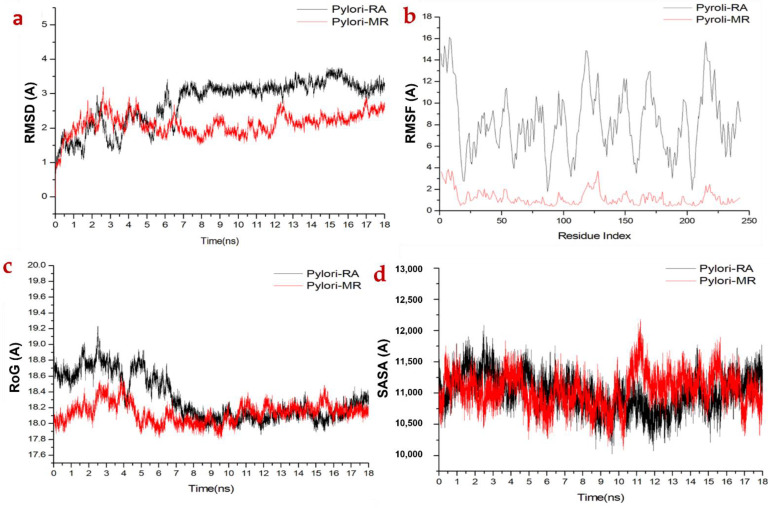
(**a**) RMSD of Cα atoms of the protein backbone atoms; (**b**) RMSF of each residue of the protein backbone Cα atoms; (**c**) ROG of Cα atoms of protein residues; (**d**) solvent accessible surface area (SASA) of the backbone atoms relative to the starting.

Molecular dynamic (MD) simulations were carried out to investigate the inhibition performance and interactions of the potential ligands with MRSA peptidoglycan transpeptidases and *H. pylori* urease targets. The validation of system stability is essential to trace disrupted motions and avoid artifacts that may arise during the simulation course. In this study, Root-Mean-Square Deviation (RMSD) was calculated to measure the systems’ stability during the 18 ns simulations (Figure 4). The recorded average RMSD values for all frames of systems MRSA-RA and MRSA-MR were 2.89 Å and 2.22 Å, respectively. Additionally, average RMSD values of 3.02 Å and 2.41 Å were observed for *H pylori*-RA and *H pylori*-MR, respectively (Figure 4a and Figure 5a). These results revealed that MR complex systems acquired a relatively more stable conformation compared to the RA system. In general, the RMSD profile of all four complexes’ systems reveals favorable interaction of MR with the targeted receptors, which are thereby stabilized during dynamics.

The assessment of protein structure flexibility upon ligand binding is essential in probing residue behavior and their association with the ligand during MD simulation [35]. MRSA peptidoglycan transpeptidases and *H. pylori* urease residual fluctuations were evaluated using the Root-Mean-Square Fluctuation (RMSF) algorithm to assess the effect of inhibitor binding towards the respective targets over 18 ns simulations. The computed average RMSF values were 4.76 Å and 1.77 Å for MRSA-RA and MRSA-MR, respectively, while 8.25 Å and 1.193 Å were recorded for *H pylori*-RA and *H pylori*-MR, respectively. Overall residual fluctuations of individual systems are represented in Figure 4b and Figure 5b. These values revealed that MR has the lower residue fluctuation during MRSA and *H. pylori* inhibition.

ROG was calculated to measure the overall compactness of the system as well as stability upon ligand binding during MD simulation [36,37]. The average Rg values were 37.25 Å and 37.23 Å for MRSA-RA and MRSA-MR, respectively, and 18.62 Å and 18.18 Å for *H. pylori*-RA and *H. pylori*-MR, respectively (Figure 4c and Figure 5c). The observed behavior suggested that MR compounds exhibited a highly rigid structure against MRSA.

The compactness of the protein hydrophobic core was investigated by evaluating the solvent accessible surface area (SASA) of the protein. This was accomplished by measuring the surface area of the protein visible to the solvent, which is significant for the stability of biomolecules [38]. The average SASA values were 27,090.30 Å and 26,961.69 Å for MRSA-RA and MRSA-MR, respectively, while 11,247.48 Å and 11,044.57 Å were observed for *H pylori*-RA and *H pylori*-MR, respectively (Figure 4d and Figure 5d). The SASA result combined with the observation from RMSD, RMSF and ROG calculations further confirmed that the MR complex system was able to remain intact inside the narrow groove of the active site during the whole course of MDS with a high stability during the simulation.

#### 2.3.2. Mechanism of Binding Interactions Based on Binding Free Energy Calculation

The binding of a ligand to a specific pharmacological target provides the structural basis for that ligand’s activity. Therefore, predicting protein–ligand binding affinities based on free binding free energy calculations is an attractive approach to discover new protein inhibitors. The MM-GBSA program in AMBER18 was used in calculating the binding free energies by extracting snapshots from the trajectories of the compounds. As shown in Table 3, all the calculated energy components (except ΔGsolv) gave high negative values, thus indicating favorable interactions.

Binding free energy (ΔGbind) values −11.22 and −40.54 Kcal/mol were obtained for the interactions of MRSA with compounds RA and MR, respectively. The ΔGbind estimated for *H. pylori* inhibition with the same ligands were −23.36 and −41.54 kcal/mol, respectively. This indicates a more favorable binding of MR towards MRSA and *H. pylori* compared to RA. It was fascinating to observe that the calculated binding free energies gave a good order regarding the experimentally determined MIC values.

A close look at the individual contribution of energy revealed that the more positive van der Waals energy components drove both RA and MR compound interactions with the MRSA and *H. pyrlori* enzyme, resulting in the observed binding free energies. Substantial binding free energy values were observed in the gas phase for all the inhibition process with values up to −73.24 and −34.17 kcal/mol, respectively (Table 3).

#### 2.3.3. Identification of the Critical Residues Responsible for Inhibitor Binding

**Figure 6:**
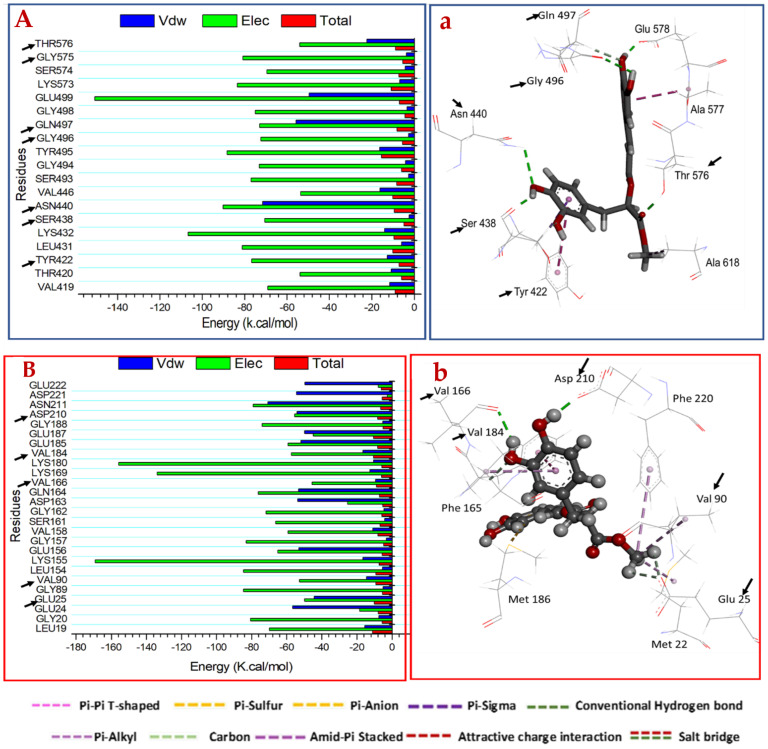
Per-residue decomposition plots showing the energy contributions to the binding and stabilization of MR at the catalytic active site of (**A**) peptidoglycan transpeptidases and (**B**) *H. pylori* urease receptor. Corresponding inter-molecular interactions are shown in (**a**) peptidoglycan transpeptidases and (**b**) *H. pylori* urease.

To obtain more insights on key residues involved in the inhibition of MRSA peptidoglycan transpeptidases and *H. pylori* urease, the total energy involved when MR bind these enzymes was further decomposed into the involvement of each site residue. As shown in Figure 6, the major favorable contribution of MR to peptidoglycan transpeptidases binding was predominantly observed from residues Tyr 422 (−12.73 kcal/mol), Lys 432 (−14.08 kcal/mol), Ser 438 (−2.34 kcal/mol), Asn 440 (−71.55 kcal/mol), Tyr 495 (−16.344 kcal/mol), Gly 496 (−2.649 kcal/mol), Gln 497 (−55.62 kcal/mol) and Thr 576 (−22.33 kcal/mol). In *H. pylori* urease residues, Leu 19 (−15.59 kcal/mol), Glu 25 (−56.60 kcal/mol), Val 90 (−14.47 kcal/mol), Val 166 (−9.45 kcal/mol), Val 184 (−16.61 kcal/mol), Asp 210 (−53.96 kcal/mol) and Asn 211 (−70.74 kcal/mol) were major contributors.

**Figure 7:**
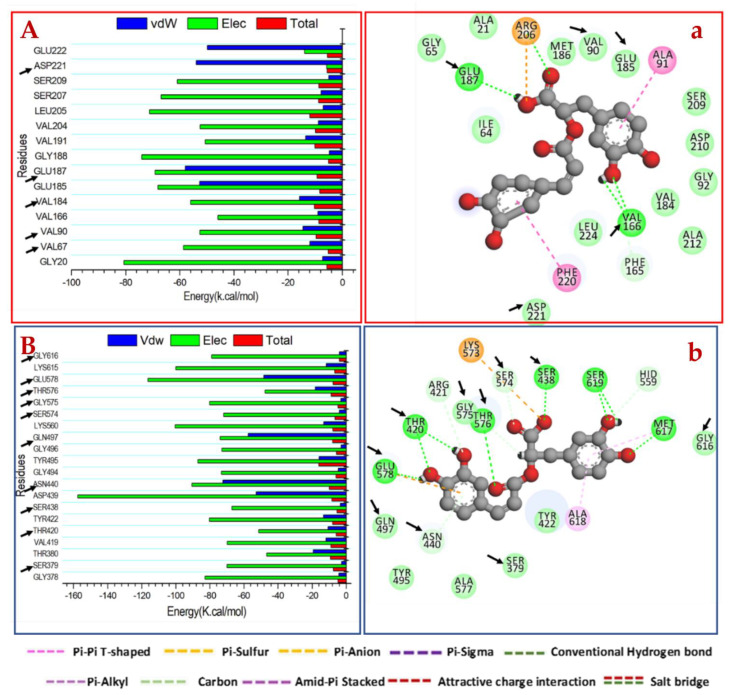
Per-residue decomposition plots showing the energy contributions to the binding and stabilization of RA at the Catalytic active site of (**A**) peptidoglycan transpeptidases and (**B**) *H. pylori* urease receptor. Corresponding inter-molecular interactions are shown in (**a**) peptidoglycan transpeptidases and (**b**) *H. pylori* urease.

On the other hand, the major favorable contribution of peptidoglycan transpeptidase inhibition by RA was predominantly observed from residues Ser 379 (−2.66 kcal/mol), Thr 420 (−10.65 kcal/mol), Ser 438 (−3.421 kcal/mol), Asn 440 (−72.29 kcal/mol), Gln 497 (−57.48 kcal/mol), Thr 576 (−18.158 kcal/mol) and Gly578 (−48.20 kcal/mol). Residues Val 90 (−14.47kcal/mol), Val 184 (−8.941 kcal/mol),), Glu 185 (−52.68 kcal/mol), Glu 187 (−57.921 kcal/mol) and Asp 221 (−53.918 kcal/mol) contributed to the *H. pylori* urease (Figure 7).

Figure 6A illustrates that MRSA peptidoglycan transpeptidase catalytic binding site residues Ser 438, Asn 440 and Glu 578 formed stable hydrogen bonds with the 3,4-dihydroxyphenyl group of MR, thereby establishing the attraction of the negatively charged residues to the positively charged hydroxyl group of the 3,4-dihydroxyphenyl groups. On the other hand, the hydroxyl groups of 3,4-dihydroxyphenyl of RA formed a hydrogen bond interaction with Thr 420, Met 617 and Ser 619 (Figure 7A). The pharmacophoric hot spot Glu 578 residue formed a hydrogen bond donor interaction with MR and RA at distances of 2.52 Å and 2.69 Å, respectively. This phenomenon showed that Glu 578 is an important feature for binding at the observed site on both ligands. A total number of 9 residues was found at the binding interface of both ligands’ interactions.

Provided in Figure 6B are the *H. pylori* urease interacting residues at the catalytic binding site with Asp 210 and Val 166 forming stable hydrogen bonds with the 3,4-dihydroxyphenyl group of MR. The 3,4-dihydroxyphenyl ring of RA formed a stable hydrogen bond with the Val 166 to produce a negative binding free energy of −8.94 kcal/mol, indicating its significance in binding (Figure 7B). Eighteen (in MR) and nineteen (in RA) residues were observed to be actively involved as major interacting moieties required for optimum binding of the ligands to *H. pylori* urease.

## 3. Discussion

Phytotherapy has received considerable attention in the scientific field based on its folk use in treating various diseases [39]. A growing number of studies have been conducted to discover new horizons of biological activities for medicinal plants and their active metabolites. Antimicrobial studies have had a large share of this research [39]. Among the various pathogenic micro-organisms, methicillin-resistant *S. aureus* (MRSA) and *H. Pylori* have received significant attention. MRSA plays a chief role in the epidemiology and pathogenesis of infections in humans, especially in the skin and soft tissues [5,6], while gastritis is always expected in cases of *H. pylori* infections, which affect 70.1% of the population in Africa [7]. 

From an ethnobotanical point of view, *Cordia africana* Lam. is one of the most widely recognized indigenous plants in Africa [1]. The stem bark is used in the treatment of wounds, skin infections and gastritis [4].

In the current investigation, the authors tried to rationalize the traditional use of *Cordia africana* Lam. stem bark based on the phytochemical and antibacterial properties against these two pathogenic microorganisms: methicillin-resistant *S. aureus* (MRSA) and *H. pylori.* Four compounds have been first isolated and identified as *α*-amyrin (1) and *β*-sitosterol (2), rosmarinic acid (RA) (3) and methyl-rosmarinate (MR) (4). The occurrence of RA and MR was confirmed by HPLC analysis, in which MR was the major identified phenolic acid (727.66 ppm) in addition to ten other phenolic acids and five flavonoids.

The antibacterial activity of the ethanolic extract and the isolated RA and MR against MRSA and *H. pylori* using the broth microdilution method demonstrated that MR showed the best bacterial inhibitory activity against MRSA and *H. pylori* with MIC 7.81 ±1.7 and 31.25 ± 0.9 μg/mL, respectively, followed by RA (MIC 31.25 ± 0.6 and 125 ± 0.58 μg/mL, respectively) compared to vancomycin (MIC 0.98 ± 1.6 μg/mL) and clarithromycin (MIC 1.95 ± 0.8 μg/mL). Vancomycin and clarithromycin were chosen as positive controls due to their actual use as potent antibiotics in MRSA and *H. pylori* eradication remedy protocols, respectively [40]. Although the results of the MR and RA compounds are far from those of the standards, they could be considered significant against these two pathogenic bacteria and in coincidence with previous studies on bioactive compounds reported to possess antibacterial activities against MRSA [41] and *H. pylori* [39]. The classification criteria for significance of the antibacterial results are stringent [42,43]. Several studies considered the plant extracts to be significantly active as antibacterial when their MIC values were ≤100 µg/mL, active for MIC values 100 to 800 µg/mL and inactive for values >800 µg/mL [41,42,44], but several studies decide to keep the level of 10–50 µg/mL for acceptable activity of bioactive metabolites [45]. In this context, MR was significantly active against MRSA and *H. pylori*, RA was active against MRSA and moderately active against *H. pylori*, while the ethanolic extract showed moderate activity against MRSA.

In the molecular docking study, two enzymes, peptidoglycan transpeptidases and urease enzymes, were chosen according to the reported literature [14,19,34] and their importance to MRSA and *H. pylori*, respectively. Peptidoglycan transpeptidase is responsible for the resistant behavior of MRSA to *β*-lactam antibiotics [15,46]. Furthermore, urease is an essential enzyme for the virulence of *H. pylori*. The bacteria produces high concentrations to be able to buffer and persist in the acidic pH conditions of the gastric juice [23].

From the molecular docking study, it was noticed that MRSA peptidoglycan transpeptidase catalytic binding site residues Ser 438, Asn 440 and Glu 578 formed a stable hydrogen bond with the planar 3,4-dihydroxyphenyl group of MR, thereby establishing an attraction of the negatively charged residues to the positively charged hydroxyl group of the 3,4-dihydroxyphenyl groups and allowing the MR to extend across the narrow active site groove. Furthermore, the binding mode study revealed that the 3,4-dihydroxyphenyl ring was a key player in its stabilization inside the active site, where it was sandwiched between Thr-618 and Tyr-422. The relevance of this planar contact is also supported by the clustering of anti-MRSA-lactam structures [47].

On the other hand, the hydroxyl groups of 3,4-dihydroxyphenyl of RA formed a hydrogen bond interaction with Thr 420, Met 617 and Ser 619. The pharmacophoric hot spot Glu 578 residue formed a hydrogen bond donor interaction with MR and RA at a distance of 2.52 Å and 2.69 Å, respectively. This phenomenon showed that Glu 578 is an important feature for binding at the observed site on both ligands. A total number of 9 residues was found at the binding interface of both ligands’ interaction.

In the same context, the *H. pylori* urease interacting residues at the catalytic binding site with Asp 210 and Val 166 form stable hydrogen bonds with the 3,4-dihydroxyphenyl group of MR. The 3,4-dihydroxyphenyl ring of RA forms a stable hydrogen bond with the Val 166 to produce a negative binding free energy of −8.94 kcal/mol, indicating its significance in binding. Eighteen (in MR) and nineteen (in RA) residues were observed to be actively involved as major interacting moieties required for optimum binding of the ligands to *H. pylori* urease. Hence, we conclude that the increasing in the nucleophilicity by the methyl group leads to an increase in their accessibility to the active site and in turn its bioactivity. Such structural information and the binding mode study can help in the design of more potent non-covalent inhibitors.

Furthermore, antibacterial activity of the identified phenolics through HPLC analysis has been extensively studied against MRSA, such as caffeic acid [48], quercetin [49] and Kampferol [50]. It was previously reported that *β*-sitosterol had potential antibacterial action illustrated through a molecular docking study [51]. We believe our study might spotlight the value of *Cordia africana* as a rich source of secondary metabolites and a promising candidate with potential antibacterial activity. Further in vivo studies are required to prove the ethnobiological use of the plant for treatment of gastritis and wound healing.

## 4. Materials and Methods

### 4.1. Plant Material and Extraction

The stem bark of *Cordia africana* Lam. was harvested in October 2017 from Basunga, Gadarif State, Sudan. A voucher sample (17.12.16.3) was kindly identified by Dr. Haider Abd-El-Qader Assistant Professor of plant taxonomy, medicinal and aromatic plants research institute, Sudan and kept in the herbarium of the department of pharmacognosy, faculty of pharmacy, Cairo University. Five hundred g of the powdered air-dried stem bark were extracted by cold maceration in 70% ethanol until exhaustion, and the solvent was evaporated under reduced pressure, yielding 72 g. The dried extract was suspended in 300 mL of distilled water and partitioned with CH_2_Cl_2_ and *n*-butanol saturated with water (10 × 500 mL, each). The dried fractions yielded 15 and 24 g of dried residues of methylene chloride and *n*-butanol, respectively.

### 4.2. Chemicals and Reagents

Different chromatographic techniques were used—viz., vacuum liquid chromatography (VLC) using silica gel H (Merck, Darmstadt, Germany), column chromatography (CC) using silica gel 60 (70–230 mesh ASTM; Fluka, Steinheim, Germany), and Sephadex LH-20 (Pharmacia, Stockholm, Sweden), thin-layer chromatography (TLC) using precoated silica gel F_254_ (Fluka). The following solvent systems were utilized for TLC: S_1_, CH_2_Cl_2_; S_2_, CH_2_Cl_2_-CH_3_OH (9.5:0.5) and S_3_, CH_2_Cl_2_-CH_3_OH-formic acid (9:1:0.1), and spots were visualized by examination under UV light (254 and 365 nm), by spraying with *p*-anisaldehyde and aluminum chloride reagents. ^1^H-NMR (400 MHz) and ^13^C-NMR (100 MHz) measurements were performed utilizing Bruker 400 MHz & 100 MHz nuclear magnetic resonance spectrometer using CDCl_3_ and CD_3_OD for sample preparation.

### 4.3. Determination of Total Phenolic Acid and Flavonoid Contents

The total phenolic acid content of the ethanolic extract of stem bark was estimated adopting the method described by Saboo et al. (2010) [52], using a Shimadzu spectrophotometer (UV-1650PC). A stock solution (1 mg/mL) was prepared by dissolution of 10 mg of the ethanolic extract in 10 mL of 95% ethanol. An aliquot of 100 μL of plant extract was combined with 500 μL Folin-Ciocalteu reagent (freshly diluted 1/10 with distilled water) and 1.5 mL of Na_2_CO_3_ (20%). After 30 min of incubation with slight shaking in the dark at ambient temperature, the absorbance was computed at λ_max_ 765 nm against blank, and the total phenolic acid content was calculated in µg of gallic acid equivalents (µg GAE/mg of dried ethanolic extract).

The total flavonoid content was evaluated by the aluminum chloride colorimetric method [53] using quercetin as standard. A stock solution (1 mg/mL) was prepared by dissolution of 10 mg of the ethanolic extract in 10 mL of 95% ethanol. An aliquot (0.8 mL) of ethanolic extract (1 mg/mL) was combined with 1.5 mL methanol, 0.1 mL aluminum chloride, 0.1 mL potassium acetate solution and 2.8 mL distilled water. The absorbance was recorded after 30 min at λ_max_ 415 nm against a blank. The total flavonoid content was calculated in µg of quercetin equivalent (µg QE/mg dried ethanolic extract).

### 4.4. Phytochemical Isolation of Compounds

The methylene chloride extract (15 g) was packed on vacuum liquid chromatography (VLC) column (5 × 55 cm, silica gel H, 120 g). Gradient elution was applied using mixtures of *n*-hexane/CH_2_Cl_2_ and CH_2_Cl_2_/ethyl acetate. Fractions of 100 mL each were collected and visualized on precoated TLC silica gel F_254_ plates using S_1_, CH_2_Cl_2_ as the solvent system. Fractions (20% and 25% CH_2_Cl_2_/Hexane) were refractionated on different columns packed with silica gel 60 and eluted with CH_2_Cl_2_, two pure compounds 1 (50 mg, white needle crystals, R_f_ 0.49 in S_1_) and 2 (45 mg, white needle crystals, R_f_ 0.23 in S_1_) were isolated.

The *n*-butanol fraction (24 g) was fractionated on VLC silica gel column (5 × 20 cm, 200 mg). Gradient elution was applied using CH_2_Cl_2_/EtOAc and EtOAc/CH_3_OH mixtures of increasing polarity. The collected fractions (200 mL) were visualized on TLC precoated plates using S_2_, CH_2_Cl_2_-CH_3_OH (9.5:0.5) and S_3_, CH_2_Cl_2_-CH_3_OH-formic acid (9:1:0.1). Similar fractions were collected and evaporated under reduced pressure, and three main fractions A, B and C, were produced. Fraction A (1.2 g, 20–35% EtOAc in CH_2_Cl_2_), was purified on sephadex LH-20 using CH_3_OH-H_2_O (1:1) as solvent system. Two major spots were observed using TLC and S_3_ as solvent system. Purification was performed using silica column (20 × 1.5 cm), eluted with CH_2_Cl_2_ and increasing polarity with CH_3_OH. Similar fractions were pooled together, yielding two main fractions, A and B. Fraction A showed one spot having R_f_ = 0.8 cm in S_3_, yielding compound 3 (30 mg) as yellow crystals in methanol after cooling, while fraction B showed another spot with R_f_ 0.4 cm in S_3_, yielding compound 4 (35 mg) as yellow crystals in methanol after cooling.

### 4.5. HPLC Profiling

#### 4.5.1. Sample Preparation

A sample of 75 μg/mL was prepared by dissolving the ethanolic extract of stem bark in methanol, which was then ultra-sonicated for 10 min and filtered through a microfilter (0.4 µm pore size) before injection.

#### 4.5.2. Standard Solution

One mg of each available phenolic reference standard (*p*-Hydroxybenzoic, syringic, ferulic, *o*-coumaric, cinnamic and rosmarinic acids, methyl rosmarinate, quercetin, naringenin, kaempferol, myricetin and rutin) was mixed and dissolved in methanol to yield a stock solution of 1 mg/mL. A calibration curve was prepared for each standard by extended dilution of the stock solution with methanol. The injected volume was (20 μL). All standards were purchased from sigma (USA).

#### 4.5.3. Analytical Condition

HPLC analysis was performed following the Agilent Application guidance, publication number 5991-3801EN [54] on an Agilent 1260 infinity series (Agilent Technologies, Santa Clara, CA, USA) supplied with Quaternary pump, a Kinetix EVO C_18_ column (5 µm, 4.6 × 100 mm) operated at 30 °C. Separation was achieved at a solvent flow rate of 1 mL/min, ultraviolet detection set at λ_max_ 284 nm and the injection volumes were 20 µL. The mobile phase was composed of a mixture of (A) 0.2% H_3_PO_4_ in HPLC grade water (*v/v*), (B) methanol and (C) acetonitrile in a gradient linear mode. Linearity was confirmed by obtaining five-point calibration curves within a concentration range of 0.006 to 1 mg/mL.

### 4.6. Determination of Antibacterial Activities

Antibacterial activity of *Cordia africana* Lam. ethanolic stem bark extract and the pure isolated RA and MR was determined using the broth microdilution method complying with the Clinical and Laboratory Standards Institute guidelines [55] to estimate the concentrations required, inhibiting 90% and 50% of bacterial growth (MIC_90_ and MIC_50_).

Methicillin-resistant *Staphylococcus aureus* (MRSA) ATCC 25923 and *Helicobacter pylori* ATCC 43504 strains were obtained from the Regional Center of Mycology & Biotechnology, Cairo, Egypt.

Methicillin-resistant *S. aureus* (MRSA) was grown in Muller–Hinton broth (MHB; Merck) for one day, and a sterile saline was used to standardize the turbidity of the microbial suspension to 0.5 McFarland scale (approximately 1–2 × 10^8^ CFU/mL). Methicillin-resistance of *S. aureus* was assessed by susceptibility testing.

*Helicobacter pylori* was grown in Brucella broth (Sigma-Aldrich, St Louis, MO, USA) for 72 h and was composed of 5% (*v/v*) horse blood and 10% (h/h) fetal bovine serum (Sigma-Aldrich) incubated under microaerophilic atmosphere (5% O_2_, 10% CO_2_ and 85% N_2_) at 37 °C. Then, a sterile saline was employed to standardize the microbial suspension turbidity to 0.5 McFarland scale (approximately 1–2 × 10^7^ CFU/mL).

Ten concentrations of each of the stem bark ethanolic extracts, RA and MR (two-fold dilution 1.95–1000 μg/mL), were separately dissolved in 10% dimethyl sulfoxide (DMSO) and preserved at −20 °C. The plates were incubated at 37 °C for 72 h in microaerophilic ambiant.

After incubation, the MIC was determined according to the coloration generated by the reaction with 3-(4,5-dimethyl-thiazol-2-yl)-2,5-diphenyltetrazolium bromide (MTT) (40 μL, 0.05 mg/mL, Merck, Darmstadt, Germany) poured into each well and left for 30 min, where a change from yellow to purple indicated the cell viability. Clarithromycin and vancomycin were used as reference drugs and as positive controls for *Helicobacter pylori* and MRSA, respectively. DMSO served as a negative control. The percentage inhibition of bacterial growth by different concentrations of each sample was estimated (average of triplicate measurements) and a dose-response curve was drawn.

### 4.7. Molecular Docking Analysis

Molecular docking studies were performed with the known Methicillin-resistant *Staphylococcus aureus* (MRSA) peptidoglycan transpeptidases and *H. pylori* urease molecular targets. These targets were chosen based on the Pharm Mapper Server screening [56]. A preliminary molecular docking simulation was performed to assess the putative binding mode of RA and MR with the proposed targets. Then, molecular dynamic simulations were conducted to investigate the inhibitory mechanism as well as selectivity impact of these potential inhibitors toward these target receptors.

Computational modeling of the studied ligands and enzyme-ligand systems revealed that lead compounds RA and MR can potentially inhibit both Methicillin-resistant *Staphylococcus aureus* (MRSA) peptidoglycan transpeptidases and *H. pylori* urease, thereby creating a pathway via the use of a double-target approach in antibacterial treatment.

#### 4.7.1. System Preparation

The X-ray crystal structures of MRSA peptidoglycan transpeptidases and *H. pylori* urease were retrieved from the protein data bank with codes 4DKI and 1E9Y, respectively [57,58]. These structures were then prepared for molecular dynamics (MD) studies using UCSF Chimera [59]. The missing residues were modeled using MODELLER 9.19 (Webb and Sali 2014) integrated with Chimera software. RA and MR compounds were drawn using ChemBioDraw Ultra 12.1. Hydrogen atoms were added to the ligand and removed from the receptor. Altogether, all four prepared systems were subjected to 18 ns MD simulations as described in the simulation section.

#### 4.7.2. Molecular Dynamic (MD) Simulations

The integration of Molecular dynamic (MD) simulations in biological systems’ study enable the exploration of the physical motion of atoms and molecules that can be difficult to access by any other means [60]. The insight extracted from performing this sort of simulation provides an intricate perspective into the biological systems’ dynamical evolution, such as conformational changes and molecule association [60]. The MD simulations of all systems were performed using the GPU version of the PMEMD engine present in the AMBER 18 package [61].

Each compound’s partial atomic charge was generated with ANTECHAMBER, utilizing the General Amber Force Field (GAFF) protocol [62]. Each system was implicitly solvated within an orthorhombic box of TIP3P water molecules within 10 Å of any box edge, performed by the Leap module of the AMBER 18 package. Neutralization of each system was further implemented via Na^+^ and Cl^−^ counter ions integrated with the Leap module.

An initial minimization of each system was performed for 2000 steps in the presence of an applied restraint potential of 500 kcal/mol, followed by a full minimization of 1000 steps carried out by conjugate gradient algorithm omitting restraints.

Each system was gradually heated during the MD simulation from 0 K to 300 K for 500 ps, ensuring that all systems maintained a fixed number of atoms with a fixed volume. The system’s solutes were imposed with a potential harmonic restraint of 10 Kcal/mol and a collision frequency of 1 ps. The ensuing heating equilibration of each system was performed for 500 ps at a constant temperature of 300 K. The number of atoms and pressure within each system for each production simulation were kept constant to mimic an isobaric-isothermal (NPT) ensemble, with the system’s pressure being maintained at 1 bar using the Berendsen barostat [63]. Each system was MD simulated for 18 ns. In each simulation, the SHAKE algorithm was employed to constrain the hydrogen bond atoms. The step size of each simulation was 2 fs and integrated an SPFP precision model. The simulations coincided with an isobaric-isothermal ensemble (NPT), with randomized seeding, constant pressure of 1 bar, a pressure-coupling constant of 2 ps, a temperature of 300 K and Langevin thermostat with a collision frequency of 1 ps.

#### 4.7.3. Post-MD Analysis

The trajectories generated after MD simulations were each saved every 1 ps, followed by analysis using the CPPTRAJ [64] module implemented in the AMBER18 suite. All plots and visualizations were completed using the Origin [65] data analysis tool and Chimera [59], respectively.

#### 4.7.4. Thermodynamic Calculation

To estimate the binding interaction of the studied compounds toward the MRSA peptidoglycan transpeptidases and *H. pylori* urease receptors, binding free energy was computed using molecular mechanics integrated with the Poisson–Boltzmann or generalized Born and surface area continuum solvation (MM/PBSA and MM/GBSA) approach [66]. MM/GB-SA and MM/PB-SA rely on the ligand–protein complex molecular simulations to compute rigorous statistical–mechanical binding free energy within a specified force field [67].

Binding free energy averaged over 180 snapshots extracted from the entire 18 ns trajectory. The estimation of the change in binding free energy (ΔG) for each molecular species (complex, ligand and receptor) can be represented as follows [68]:

(1) $$∆G_{\mathrm{bind}}=G_{\mathrm{complex}}-G_{\mathrm{receptor}}-G_{\mathrm{ligand}}$$

(2) $$∆G_{\mathrm{bind}}=E_{\mathrm{gas}}+G_{\mathrm{sol}}-\mathrm{TS}\;$$

(3) $$E_{\mathrm{gas}}=E_{\mathrm{int}}+E_{\mathrm{vdw}}+E_{\mathrm{ele}}\;$$

(4) $$G_{\mathrm{sol}}=G_{\mathrm{GB}}+G_{\mathrm{SA}}$$

(5) $$G_{\mathrm{SA}}=\gamma \mathrm{SASA}\;$$

The terms E_gas_, E_int_, E_ele_ and E_vdw_ symbolized the gas-phase energy, internal energy, Coulomb energy and van der Waals energy, respectively. The E_gas_ was directly assessed from the FF14SB force field terms. Solvation free energy (G_sol_) was evaluated from the energy involvement from the polar states (G_GB_) and non-polar states (G). The non-polar solvation free energy (G_SA_) was determined from the Solvent Accessible Surface Area (SASA) [69] using a water probe radius of 1.4 Å. In contrast, the polar solvation (G_GB_) contribution was assessed by solving the GB equation. Items S and T symbolize the total entropy of the solute and temperature, respectively. Each residue contributes to the total binding free energy obtained at the predicted active site by carrying out per-residue energy decomposition at the atomic level using the MM/GBSA method in AMBER 18 [66].

## 5. Conclusions

In the current investigation, the ethnobotanical use of the stem bark of *Cordia africana* Lam. was rationalized through an investigation of the antibacterial activity against the two pathogenic bacteria MRSA and *H. pylori*. The binding mode of compounds RA and MR with MRSA peptidoglycan transpeptidases and *H. pylori* urease were simulated using different computational tools. This study addressed what might be beneficial in the molecular understanding of these two potentially active ligands, thereby contributing to antibacterial drug discovery.

## Figures and Tables

**Table 1:** Identified phenolic compounds in *Cordia africana* Lam. stem bark ethanolic extract by HPLC at λ_max_ 284 nm.

Peak No.	Identified Phenolic Compounds	R_t_	Concentration (ppm)
1.	*p*-hydroxy benzoic acid	9.08	124.68
2.	Caffeic acid	11.72	8.95
3.	Syringic acid	12.39	22.44
4.	Ferulic acid	15.99	14.47
5.	Rutin	18.24	499.63
6.	*o*-Coumaric acid	19.76	23.07
7.	Myricetin	21.14	452.86
8.	Methyl rosmarinate	22.10	727.66
9.	Quercetin	25.25	239.75
10.	Rosmarinic acid	25.42	138.18
11.	Naringenin	26.64	1971.68
12.	Kaempferol	27.59	943.38

**Table 2:** Mean MIC (μg/mL ± SD) values of *Cordia africana* Lam. stem bark ethanolic extract and the isolated rosmarinic acid (RA) and methyl rosmarinate (MR) against *H. pylori* and methicillin-resistant *Staphylococcus aureus* (MRSA).

	*H. pylori*		MRSA	
	MIC_90_	MIC	MIC_90_	MIC
Ethanolic extract	382.8 ± 0.96	1000 ± 0.55	238 ± 1.9	500 ± 0.8
Rosmarinic acid (RA)	87.7 ± 0.85	125 ± 0.58	15.63 ± 0.96	31.25 ± 0.6
Methyl rosmarinate (MR)	14.4 ± 1.7	31.25 ± 0.9	5.14 ± 2.1	7.81 ± 1.7
Vancomycin	-	-	0.46 ± 1.8	0.98 ± 1.6
Clarithromycin	0.7 ± 1.3	1.95 ± 0.8	-	-

All determinations were carried out in triplicate.

**Table 3:** Summary of the energy binding calculated for methyl rosmarinate (MR) and rosmarinic acid (RA) against methicillin-resistant *Staphylococcus aureus* (MRSA) and *H. pylori*.

Energy Components (Kcal/mol)						
**MRSA**						
**Complex**	**MIC**	**ΔE_vdW_**	**ΔE_elec_**	**ΔG_gas_**	**ΔG_solv_**	**ΔG_bind_**
**RA**	31.25	−27.21 ± 0.22	−19.97 ± 1.49	−47.19 ± 1.40	35.96 ± 1.04	−11.22 ± 0.45
**MR**	7.81	−46.03 ± 0.06	−26.78 ± 0.08	−72.81 ± 0.09	31.35 ± 0.05	−40.53 ± 0.06
***H. pylori***						
**RA**	125	−27.20 ± 0.10	−29.12 ± 1.08	−64.31 ± 1.04	33.15 ± 1.05	−23.36 ± 0.10
**MR**	31.25	−36.97± 0.08	−43.01 ±0.19	−79.98 ± 0.14	38.44 ± 0.15	−41.54 ± 0.10

∆E_vdW_ = van der Waals energy; ∆_Eele_ = electrostatic energy; ∆G_solv_ = solvation free energy; ∆G_bind_ = calculated total binding free energy.

## Data Availability

No new data were created or analyzed in this study. Data sharing is not applicable to this article.

## Sample Availability

Samples of the compounds 1–4 are not available from the authors.

## References

[1] Yirgu A., Mohammed K., Geldenhuys C.J. (2019). Useful medicinal tree species of Ethiopia: Comprehensive review. S. Afr. J. Bot..

[2] Giday M., Teklehaymanot T., Animut A., Mekonnen Y. (2007). Medicinal plants of the Shinasha, Agew-awi and Amhara peoples in northwest Ethiopia. J. Ethnopharmacol..

[3] Alemayehu G., Asfaw Z., Kelbessa E. (2016). *Cordia africana* (Boraginaceae) in Ethiopia: A review on its taxonomy, distribution, ethnobotany and conservation status. Int. J. Bot. Stud..

[4] Tijjani R.G., Umar M.L., Hussaini I.M., Shafiu R. (2016). Anti-nociceptive activities of the ethanolic stem bark extract of *Cordia africana* (Boraginaceae) in rats and mice. Ann. Biol. Sci..

[5] Gordon R.J., Lowy F.D. (2008). Pathogenesis of Methicillin-Resistant *Staphylococcus aureus* Infection. Clin. Infect. Dis..

[6] Crawford S.E., David M.Z., Glikman D., King K.J., Boyle-Vavra S., Daum R.S. (2009). Clinical importance of purulence in methicillin-resistant *Staphylococcus aureus* skin and soft tissue infections. J. Am. Board Fam. Med..

[7] Smith S., Fowora M., Pellicano R. (2019). Infections with *Helicobacter pylori* and challenges encountered in Africa. World J. Gastroenterol..

[8] Dagnachew Y., Asteraye Y., Mensigitu T., Anza M. (2015). Extraction and Physico-chemical Characterization of *Cordia africana* Lam Seed Oil. J. Adv. Bot. Zool..

[9] Sarah T.-B. (2013). Ferric reducing antioxidant power and total phenols in *Cordia africana* fruit. Afr. J. Biochem. Res..

[10] Mohamed M.A.A. (2016). Phytochemical and Biological Studies of *Cordia africana* Family Boraginaceae Cultivated in Egypt. CU Theses.

[11] Isa A.I., Saleh M.I.A., Abubakar A., Dzoyem J.P., Adebayo S.A., Musa I., Sani U.F., Daru P.A. (2016). Evaluation of anti-inflammatory, antibacterial and cytotoxic activities of *Cordia africana* leaf and stem bark extracts. Bayero J. Pure Appl. Sci..

[12] Alhadi E.A., Khalid H.S., Alhassan M.S., Ali A.A., Babiker S.G., Alabdeen E.M.Z., Kabbashi A.S. (2015). Antioxidant and cytotoxicity activity of *Cordia africana* in Sudan. J. Med. Plant Res..

[13] Mekonnen A., Degu Y., Carlson R. (2020). Appraisal of solvent system effect on bioactivity profiling of *Cordia africana* stem bark extracts. Chem. Int..

[14] Eseonu K.C., Middleton S.D., Eseonu C.C. (2011). A retrospective study of risk factors for poor outcomes in methicillin-resistant *Staphylococcus aureus* (MRSA) infection in surgical patients. J. Orthop. Surg. Res..

[15] Triboulet S., Dubée V., Lecoq L., Bougault C., Mainardi J.L., Rice L.B., Ethève-Quelquejeu M., Gutmann L., Marie A., Dubost L. (2013). Kinetic Features of L,D-Transpeptidase Inactivation Critical for β-Lactam Antibacterial Activity. PLoS ONE.

[16] Foster T.J. (2019). Can β-Lactam Antibiotics Be Resurrected to Combat MRSA?. Trends Microbiol..

[17] Morrisette T., Alosaimy S., Abdul-mutakabbir J.C., Kebriaei R., Rybak M.J. (2020). The Evolving Reduction of Vancomycin and Daptomycin Susceptibility in MRSA—Salvaging the Gold Standards with Combination Therapy. Antibiotics.

[18] Montecucco C., Rappuoli R. (2001). Living dangerously: How *Helicobacter pylori* survives in the human stomach. Nat. Rev. Mol. Cell Biol..

[19] Uemura N., Okamoto S., Yamamoto S., Matsumura N., Yamaguchi S., Yamakido M., Taniyama K., Sasaki N., Schlemper R.J. (2001). *Helicobacter pylori* Infection and the Development of Gastric Cancer. N. Engl. J. Med..

[20] Shamsdin S.A., Alborzi A., Ghaderi A., Lankrani K.B., Pouladfar G. (2020). reza Significance of TC9 and TH9 in *Helicobacter pylori*-induced gastritis. Helicobacter.

[21] Nagata M., Toyonaga K., Ishikawa E., Haji S., Okahashi N., Takahashi M., Izumi Y., Imamura A., Takato K., Hideharu I. (2021). Helicobacter pylori metabolites exacerbate gastritis through C-type lectin receptors. J. Exp. Med..

[22] Eloff J.N., Famakin J.O., Katerere D.R.P. (2005). Isolation of an antibacterial stilbene from *Combretum woodii* (Combretaceae) leaves. Afr. J. Biotechnol..

[23] Valenzuela-Valderrama M., Cerda-Opazo P., Backert S., González M.F., Carrasco-Véliz N., Jorquera-Cordero C., Wehinger S., Canales J., Bravo D., Quest A.F.G. (2019). The *Helicobacter pylori* urease virulence factor is required for the induction of hypoxia-induced factor-1α in gastric cells. Cancers.

[24] Zhang M. (2015). High antibiotic resistance rate: A difficult issue for *Helicobacter pylori* eradication treatment. World J. Gastroenterol..

[25] Malfertheiner P., Megraud F., O’Morain C., Bazzoli F., El-Omar E., Graham D., Hunt R., Rokkas T., Vakil N., Kuipers E.J. (2007). Current concepts in the management of *Helicobacter pylori* infection: The maastricht III consensus report. Gut.

[26] Ahn Y.J., Park S.J., Lee S.G., Shin S.C., Choi D.H. (2000). Cordycepin: Selective growth inhibitor derived from liquid culture of *Cordyceps militaris* against *Clostridium* spp.. J. Agric. Food Chem..

[27] Bae E.A., Han M.J., Kim D.H. (1999). In vitro anti-*Helicobacter pylori* activity of some flavonoids and their metabolites. Planta Med..

[28] Li Y., Xu C., Zhang Q., Liu J.Y., Tan R.X. (2005). In vitro anti-*Helicobacter pylori* action of 30 Chinese herbal medicines used to treat ulcer diseases. J. Ethnopharmacol..

[29] Ustün O., Ozçelik B., Akyön Y., Abbasoglu U., Yesilada E. (2006). Flavonoids with anti-*Helicobacter pylori* activity from *Cistus laurifolius* leaves. J. Ethnopharmacol..

[30] Geller F., Schmidt C., Göttert M., Fronza M., Schattel V., Heinzmann B., Werz O., Flores E.M.M., Merfort I., Laufer S. (2010). Identification of rosmarinic acid as the major active constituent in Cordia americana. J. Ethnopharmacol..

[31] Al-Musayeib N., Perveen S., Fatima I., Nasir M., Hussain A. (2011). Antioxidant, anti-glycation and anti-inflammatory activities of phenolic constituents from cordia sinensis. Molecules.

[32] Singh R., Lawania R.D., Mishra A., Gupta R. (2010). Role of *Cordia dichotoma* seeds and leaves extract in degenerative disorders. Int. J. Pharm. Sci. Rev. Res..

[33] Nyigo V.A., Peter X., Mabiki F., Malebo H.M., Mdegela R.H., Fouche G.E., Vitus M., Nyigo A., Abiki F.M., Alebo H.M.M. (2016). Isolation and Identification of Euphol and β-Sitosterol from the Dichloromethane Extracts of Synadenium Glaucescens.

[34] Lu Y., Foo L.Y. (1999). Rosmarinic acid derivatives from Salvia officinalis. Phytochemistry.

[35] Machaba K.E., Mhlongo N.N., Soliman M.E.S. (2018). Induced Mutation Proves a Potential Target for TB Therapy: A Molecular Dynamics Study on LprG. Cell Biochem. Biophys..

[36] Pan L., Patterson J.C. (2013). Molecular Dynamics Study of Zn(Aβ) and Zn(Aβ)2. PLoS ONE.

[37] Wijffels G., Dalrymple B., Kongsuwan K., Dixon N.E. (2005). Conservation of eubacterial replicases. IUBMB Life.

[38] Richmond T.J. (1984). Solvent accessible surface area and excluded volume in proteins. Analytical equations for overlapping spheres and implications for the hydrophobic effect. J. Mol. Biol..

[39] Salehi B., Sharopov F., Martorell M., Rajkovic J., Ademiluyi A.O., Sharifi-Rad M., Fokou P.V.T., Martins N., Iriti M., Sharifi-Rad J. (2018). Phytochemicals in *Helicobacter pylori* infections: What are we doing now?. Int. J. Mol. Sci..

[40] Kocsmár É., Buzás G.M., Szirtes I., Kocsmár I., Kramer Z., Szijártó A., Fadgyas-Freyler P., Szénás K., Rugge M., Fassan M. (2021). Primary and secondary clarithromycin resistance in *Helicobacter pylori* and mathematical modeling of the role of macrolides. Nat. Commun..

[41] Xin L.Y., Min T.H., Zin P.N.L.M., Pulingam T., Appaturi J.N., Parumasivam T. (2021). Antibacterial potential of Malaysian ethnomedicinal plants against methicillin-susceptible *Staphylococcus aureus* (MSSA) and methicillin-resistant *Staphylococcus aureus* (MRSA). Saudi J. Biol. Sci..

[42] Kuete V. (2010). Potential of Cameroonian plants and derived products against microbial infections: A review. Planta Med..

[43] Cos P., Maes L., Sindambiwe J.-B., Vlietinck A.J., Berghe V.D. (2006). Bioassays for Antibacterial and Antifungal Activities. Biological Screening of Plant Constituents: Training Manual.

[44] Mabeku L.B.K., Bille B.E., Tchouangueu T.F., Nguepi E., Leundji H. (2017). Treatment of *Helicobacter pylori* infected mice with bryophyllum pinnatum, a medicinal plant with antioxidant and antimicrobial properties, reduces bacterial load. Pharm. Biol..

[45] Ríos J.L., Recio M.C. (2005). Medicinal plants and antimicrobial activity. J. Ethnopharmacol..

[46] Livermore D.M. (2000). Antibiotic resistance in staphylococci. Int. J. Antimicrob. Agents.

[47] Lim D., Strynadka N.C.J. (2002). Structural basis for the β-lactam resistance of PBP2a from methicillin-resistant *Staphylococcus aureus*. Nat. Struct. Biol..

[48] Kȩpa M., Miklasińska-Majdanik M., Wojtyczka R.D., Idzik D., Korzeniowski K., Smoleń-Dzirba J., Wasik T.J. (2018). Antimicrobial potential of caffeic acid against *Staphylococcus aureus* clinical strains. BioMed Res. Int..

[49] Ramos F.A., Takaishi Y., Shirotori M., Kawaguchi Y., Tsuchiya K., Shibata H., Higuti T., Tadokoro T., Takeuchi M. (2006). Antibacterial and antioxidant activities of quercetin oxidation products from yellow onion (*Allium cepa*) skin. J. Agric. Food Chem..

[50] Bylka W., Matlawska I., Pilewski N. (2004). Natural flavonoids as antimicrobial agents. Jana.

[51] Hoskeri J.H., Krishna V., Jignesh S., Sanjay S.T., Roshan A., Vijay S. (2012). In-silico drug designing using B-sitosterol isolated from flaveria trinervia against peptide deformylase protein to hypothesize bactericidal effect. Int. J. Pharm. Pharm. Sci..

[52] Saboo S., Tapadiya R., Khadabadi S.S., Deokate U.A. (2010). In vitro antioxidant activity and total phenolic, flavonoid contents of the crude extracts of *Pterospermum acerifolium* wild leaves (Sterculiaceae). J. Chem. Pharm. Res..

[53] Ibrahim M. (2011). Comparison of total flavanoid content of Azadirachta indica root bark extracts prepared by different methods of extraction. Res. J. Pharm. Biol. Chem. Sci..

[54] Krieger S., Sonja K., Schneider S., Krieger S. (2014). Quality Analysis of Extra Virgin Olive Oils–Part 7: Nutritive Benefits–Determination of Phenolic Compounds in Virgin Olive Oil Using the Agilent 1290 Infinity 2D-LC Solution.

[55] Clinical and Laboratory Standards Institute (2017). Performance Standards for Antimicrobial Susceptibility Testing.

[56] Liu X., Ouyang S., Yu B., Liu Y., Huang K., Gong J., Zheng S., Li Z., Li H., Jiang H. (2010). PharmMapper server: A web server for potential drug target identification using pharmacophore mapping approach. Nucleic Acids Res..

[57] Ha N.C., Oh S.T., Sung J.Y., Cha K.A., Lee M.H., Oh B.H. (2001). Supramolecular assembly and acid resistance of *Helicobacter pylori* urease. Nat. Struct. Biol..

[58] Lovering A.L., Gretes M.C., Safadi S.S., Danel F., De Castro L., Page M.G.P., Strynadka N.C.J. (2012). Structural Insights into the Anti-methicillin-resistant Staphylococcus aureus (MRSA) Activity of Ceftobiprole. J. Biol. Chem..

[59] Pettersen E.F., Goddard T.D., Huang C.C., Couch G.S., Greenblatt D.M., Meng E.C., Ferrin T.E. (2004). UCSF Chimera-A visualization system for exploratory research and analysis. J. Comput. Chem..

[60] Hospital A., Goñi J.R., Orozco M., Gelpí J.L. (2015). Molecular dynamics simulations: Advances and applications. Adv. Appl. Bioinform. Chem..

[61] Lee T.S., Cerutti D.S., Mermelstein D., Lin C., Legrand S., Giese T.J., Roitberg A., Case D.A., Walker R.C., York D.M. (2018). GPU-Accelerated Molecular Dynamics and Free Energy Methods in Amber18: Performance Enhancements and New Features. J. Chem. Inf. Model..

[62] Wang J., Wang W., Kollman P.A., Case D.A. (2006). Automatic atom type and bond type perception in molecular mechanical calculations. J. Mol. Graph. Model..

[63] Berendsen H.J.C., Postma J.P.M., Van Gunsteren W.F., Dinola A., Haak J.R. (1984). Molecular dynamics with coupling to an external bath. J. Chem. Phys..

[64] Roe D.R., Cheatham T.E. (2013). PTRAJ and CPPTRAJ: Software for processing and analysis of molecular dynamics trajectory data. J. Chem. Theory Comput..

[65] Seifert E. (2014). OriginPro 9.1: Scientific data analysis and graphing software-Software review. J. Chem. Inf. Model..

[66] Genheden S., Ryde U. (2015). The MM/PBSA and MM/GBSA methods to estimate ligand-binding affinities. Expert Opin. Drug Discov..

[67] Drissi M., Benhalima N., Megrouss Y., Rachida R., Chouaih A., Hamzaoui F. (2015). Theoretical and experimental electrostatic potential around the m-nitrophenol molecule. Molecules.

[68] Hou T., Wang J., Li Y., Wang W. (2011). Assessing the performance of the MM/PBSA and MM/GBSA methods. 1. The accuracy of binding free energy calculations based on molecular dynamics simulations. J. Chem. Inf. Model..

[69] Sitkoff D., Sharp K.A., Honig B. (1994). Accurate calculation of hydration free energies using macroscopic solvent models. J. Phys. Chem..

